# Ultrafast python‐integrated single‐entity electrochemical sensor for detecting glycated albumin

**DOI:** 10.1111/febs.70547

**Published:** 2026-04-18

**Authors:** Zihni Onur Uygun

**Affiliations:** ^1^ Faculty of Medicine, Department of Medical Biochemistry Kafkas University Kars Türkiye

**Keywords:** glycated albumin, Python, sensor, silver nanoparticle, single entity electrochemistry

## Abstract

This study reports a rapid single‐entity electrochemistry (SEE) approach for detecting glycated albumin (GA), a biomarker primarily used for short‐term glycaemic monitoring and for clinical situations in which glycated haemoglobin (HbA1c) may be unreliable. Silver nanoparticles (AgNPs) were functionalised with 4‐mercaptophenylboronic acid (MPBA) to enable cis‐diol affinity interactions with glycated moieties. The MPBA–AgNP conjugates were mixed directly with serum samples, and random nanoparticle collisions at an ultramicroelectrode‐generated chronoamperometric transient. A Python‐based workflow extracted and classified collision events using peak morphology, and quantification was performed by counting GA‐associated events within a 150 s acquisition window. The method provided a linear response from 0.1% to 50% GA (R^2^ = 0.9972 ± 0.0019) and operated in a non‐immobilised, mix‐and‐measure format. These results highlight an ultrafast SEE readout based on single‐event counting and interpretable transient features, supporting rapid GA assessment in serum matrices.

## Graphical abstract

Traditional biosensors suffer from the fact that recognition receptors are immobilised and that they denature on electrodes. Instead here we use receptors freely in solution, minimising surface compression and preserving activity to boost signal efficiency. Python‐driven processing turns the electrochemical response into a rapid, robust readout. This approach moves point‐of‐care diagnostics toward faster, low‐cost, and more reliable testing.

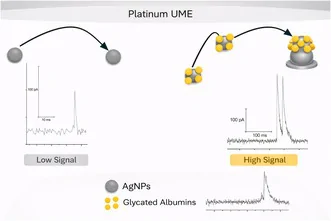

**Abbreviations**
Ag/AgCl: silver/silver chloride (reference electrode)
AgNP: silver nanoparticle
CV: cyclic voltammetry
DI: deionised (water)
DLS: dynamic light scattering
ELISA: enzyme‐linked immunosorbent assay
FTIR: Fourier‐transform infrared spectroscopy
GA: glycated albumin
Glc‐Alb: glycated albumin
Glc‐Lys: glycated lysine
HbA1c: glycated haemoglobin
HEPES: 4‐(2‐hydroxyethyl)‐1‐piperazineethanesulfonic acid
MPBA: 4‐mercaptophenylboronic acid
NBT: nitro blue tetrazolium
SEE: single‐entity electrochemistry
SPCE: single‐particle collision electrochemistry
WE: working electrode

## Introduction

Single‐entity electrochemistry (SEE) has emerged as a powerful framework for interrogating electrochemical processes at the level of individual nanoparticles, molecules, or transient events, enabling information that is obscured in ensemble‐averaged measurements [1, 2, 3]. SEE encompasses approaches such as nanoparticle collision/electrocatalytic amplification, stochastic event counting, and transient‐shape analysis, where each event provides a localised readout of particle properties, interfacial chemistry, and transport [4, 5, 6]. Over the past decade, SEE has been used to quantify particle concentrations, probe surface functionalisation, resolve heterogeneity in catalytic activity, and extract kinetic/transport parameters by analysing distributions of event amplitudes, widths and frequencies. Therefore, single entities such as nanoparticles can be easily detected by this method. The modification of the nanoparticles with selective groups such as boronic acid residues can provide selective determination of the binding targets. Parallel to these advances, boronic‐acid chemistry has been widely exploited for recognition of cis‐diol‐containing species, offering a general route to interface‐selective binding [7, 8]. However, boronic‐acid binding is inherently broad and matrix‐dependent; consequently, translating boronated recognition into fast, discriminative electrochemical readouts requires careful control of experimental conditions and quantitative evaluation of misclassification/cross‐reactivity. In this context, the present work focuses on a methodological contribution to SEE, a very rapid electrochemical acquisition coupled to single‐event feature extraction and particle‐by‐particle classification, enabling discrimination among nanoparticle states and analyte‐associated states within short measurement windows. Glycated albumin has been chosen as the target, because it is an Amadori‐reaction‐based modified protein among some of the other blood components. The biomarker can be useful for Diabetes mellitus, a chronic metabolic disorder characterised by elevated blood glucose levels, and continues to be a global health challenge with an increasing incidence worldwide [9]. Traditionally, glycated haemoglobin (HbA1c), which is produced by non‐enzymatic glycation of the proteins from different sites, has been the gold standard biomarker for diabetes diagnosis and long‐term glucose monitoring [10]. However, HbA1c possesses a notable limitation of the relatively long half‐life of approximately 120 days, which means that it reflects an extended historical average of blood glucose levels [10]. In contrast to HbA1c, glycated albumin (GA) reflects short‐term glycaemic status because albumin has a substantially shorter half‐life (≈ 2–3 weeks). This characteristic of HbA1c makes it less responsive to rapid glycaemic changes, such as those occurring in individuals with diabetes undergoing treatment adjustments or experiencing acute glucose fluctuations. In contrast, glycated albumin (GA) offers a more dynamic and responsive alternative as a biomarker for diabetes management [11]. In order to use the potential of GA as a more valuable diabetes biomarker, innovative methods for its detection are required. When comparing the existing methods for the measurement of glycated albumin, several disadvantages and differences can be observed [7, 8, 12, 13]. In clinical practice, GA measurement is usually performed under the name of fructosamine tests. Fructosamine refers to ketoamine bonds formed as a result of glycation of serum proteins (albumin) and reflects approximately 2–3 weeks of glycaemic control [14]. In classical fructosamine measurements, the reducing effect of ketoamines formed because of glycation is measured using colorimetric reagents such as nitro blue tetrazolium (NBT). Although this method is practical, it has had difficulty in routine medical examinations due to standardisation difficulties. In addition, since it measures total ketoamines, it may include not only albumin glycation but also the effects of other glycated proteins [15]. There are various laboratory techniques for more specific GA determination. Boronated affinity chromatography [16] separates glycated albumin based on the principle of the affinity of phenylboronic acid derivatives to cis‐diol groups. The glycated albumin fraction that is retained and remains on the column can then be determined by mass spectrometry or immunological methods [16]. In conclusion current methods can generally meet clinical needs in terms of sensitivity and accuracy; however, each has limitations in terms of infrastructure and time. Herein, we present a novel approach that combines the unique electrochemical properties of silver nanoparticles and phenyl boronic acid of sugar‐binding capacity. Silver nanoparticles are known for their distinctive optical and electrochemical properties, making them excellent candidates for various sensing applications [17]. Therefore, as suggested above, we have modified silver nanoparticles with boronic acid residues, which have the remarkable ability to recognise glucose molecules through the formation of boronated esters, a well‐established glucose recognition mechanism [18]. It is hypothesised that 4‐mercaptophenylboronic acid (MPBA) on AgNP surfaces via sulfur groups [19] forms reversible boronate ester interactions with cis‐diol motifs on glycated moieties (Amadori‐type structures) present on GA and other glycated serum proteins, thereby altering the single‐entity features upon nanoparticle collision. Importantly, boronic acid–cis‐diol recognition is not intrinsically GA‐specific; the chemical interaction is broadly responsive to glycated/cis‐diol–containing species. In order to avoid different species signals, it explicitly includes a Python [20] integrated system which delivers instant analytics, enables a seamless end‐to‐end workflow, generates publication‐ready visuals, and preserves transparent reproducibility for every dataset processed. This high‐throughput event detection separated in the peak‐feature space (peak current and peak width) that supports a simple and transparent decision by rule‐based thresholding, and performance characterisation in relevant matrices via spike‐in experiments and comparison against a reference method. The emphasis of this study is therefore not a diagnostic claim, but the demonstration of rapid SEE with single‐particle counting and signal clarification by isolating the target events to increase selectivity.

## Results and discussion

The nanoparticle production and conjugation stages included in this project plan were carried out. Accordingly, the CV data of the nanoparticles added to 100 μL of pH = 7.4 50 mM phosphate buffer, respectively, for the determination of the AgNPs oxidation signal on the carbon screen‐printed electrode to select an appropriate potential for chronoamperometric single‐entity measurements in phosphate media. We first recorded CVs of AgNPs in pH 7.4 phosphate buffer and identified the AgNP oxidation peak (Fig. 1). The AgNP oxidation peak position showed an ~ 200 mV shift relative to the baseline condition, which is consistent with prior reports that phosphate anions (particularly more basic phosphate species) can induce a notable cathodic shift of the AgNP oxidation peak via Ag–phosphate interactions. Accordingly, since the AgNPs oxidation signal increased by 200 mV with the concentration from the baseline signal (Fig. 1), this signal was compared as acknowledged in the study of Navolotskaya and collegues [21].

**Fig. 1:**
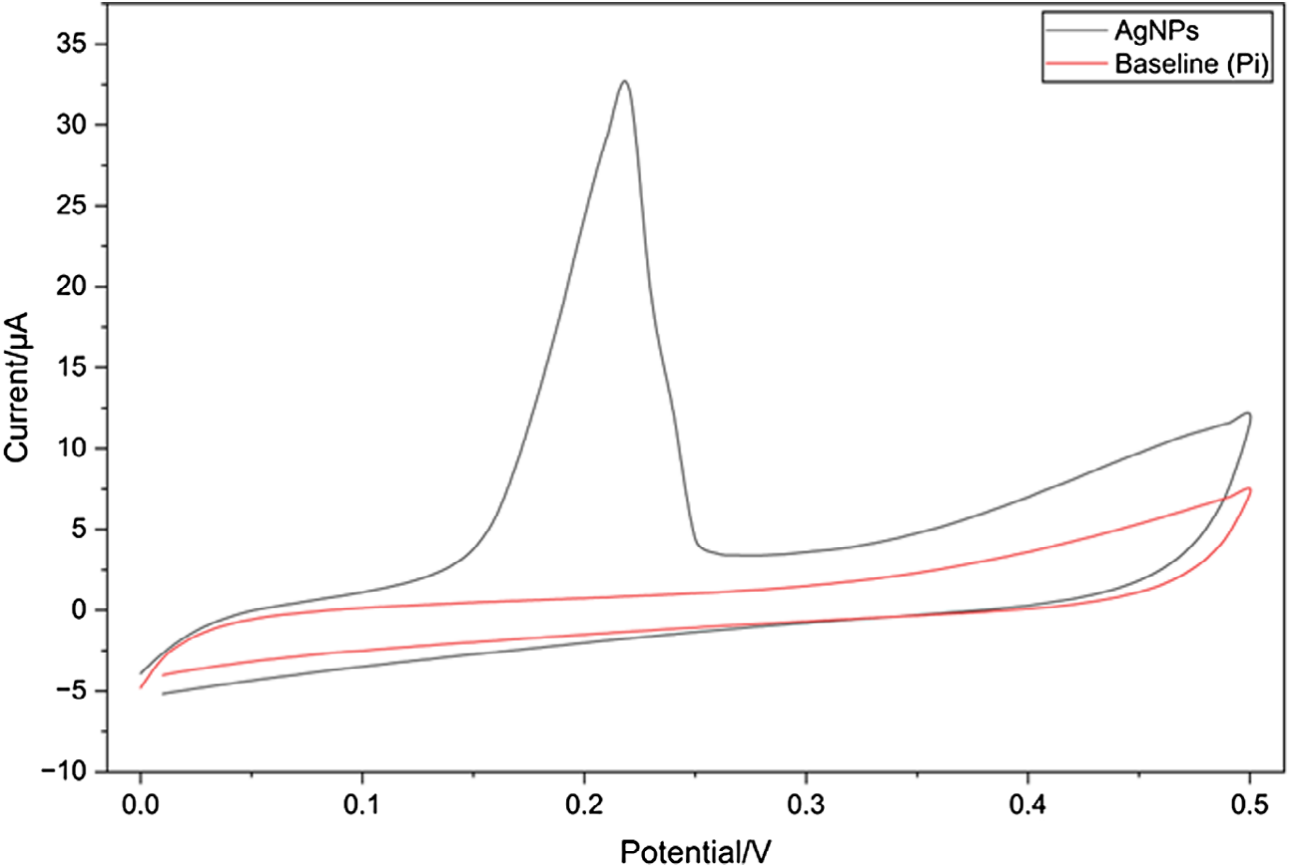
CV data applied to determine the oxidation signal of silver nanoparticles (AgNPs: 100 μL).

The particle sizes of the produced nanoparticles were measured. Accordingly, it is seen in Figs S1 and S2 that the diameters of the produced nanoparticles are ~80 nm on average. Chemical modification steps were monitored by Fourier‐transform infrared spectroscopy (FTIR) (Fig. 2) as supportive evidence of (i) boronic‐acid functionalisation on AgNPs and (ii) protein/glycation‐associated functional groups. Native albumin exhibited the expected protein bands (Amide A and Amide I/II). Upon glycation (albumin and lysine models), additional/intensified features in the 1000–1400 cm^−1^ region were observed, consistent with increased C–O/C–N contributions associated with glycation. For AgNP–4MPBA, bands characteristic of boronic‐acid vibrations were observed in the ~1180–1040 cm^−1^ region, and disappearance of the free thiol (S–H) stretch, along with low‐frequency features consistent with Ag–S bonding, supported immobilisation of 4‐MPBA onto the AgNP surface. We note that FTIR provides qualitative confirmation of functionalisation and glycation‐related groups, while analytical discrimination between GA and other cis‐diol‐containing species in this study is established primarily by the single‐entity electrochemical transient features and the interference/misclassification analysis.

**Fig. 2:**
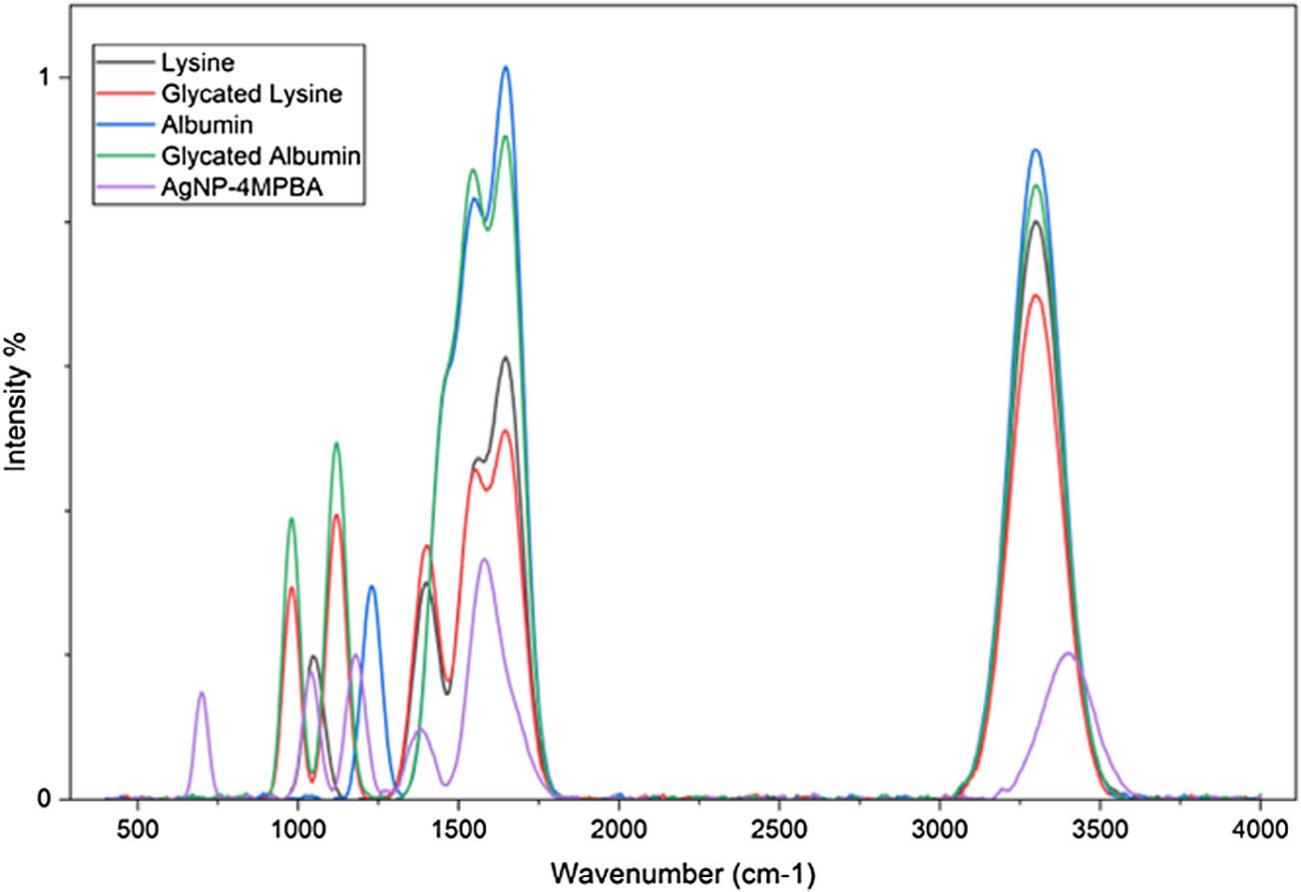
FTIR spectra of native and glycated biomolecular components together with functionalised silver nanoparticles. The spectra are color‐coded to distinguish native albumin, glycated albumin, glycated lysine, AgNPs, and AgNPs‐MPBA. Native albumin shows the expected protein‐associated bands, whereas glycated samples exhibit additional or intensified features in the 1000–1400 cm^−1^ region consistent with glycation‐related C–O/C–N contributions. AgNPs‐MPBA shows boronic‐acid‐related vibrations in the ~ 1180–1040 cm^−1^ region together with spectral changes consistent with Ag–S bonding after 4‐MPBA immobilisation.

After these processes, each material produced was tested in turn with single entity electrochemistry in phosphate buffer. In addition, commercially obtained HbA1c was added to the measurement system. The SEE method was determined as a measurement current of 200 mV with the shadowing of phosphate as in Fig. 1 [21]. The sampling time was 10 ms, and measurements were made with the current filter between 10 pA and 1 nA. The measurement continued as a measurement time of 150 s. The nano collision process of silver nanoparticles is shown in Fig. 3 with SEE (chronoamperometry). As can be seen, the measurement continued for 150 s (Fig. 3A). The peak width obtained from silver nanoparticles, and the characteristics of these peaks, are also shown in Fig. 3B. The peak currents of silver nanoparticles were measured with an average height of ~80 pA. Accordingly, the signals generated by the impact of non‐uniform nanoparticles on the surface differed, and the peak heights generated by the impact of each nanomaterial of different sizes on the surface differed.

**Fig. 3:**
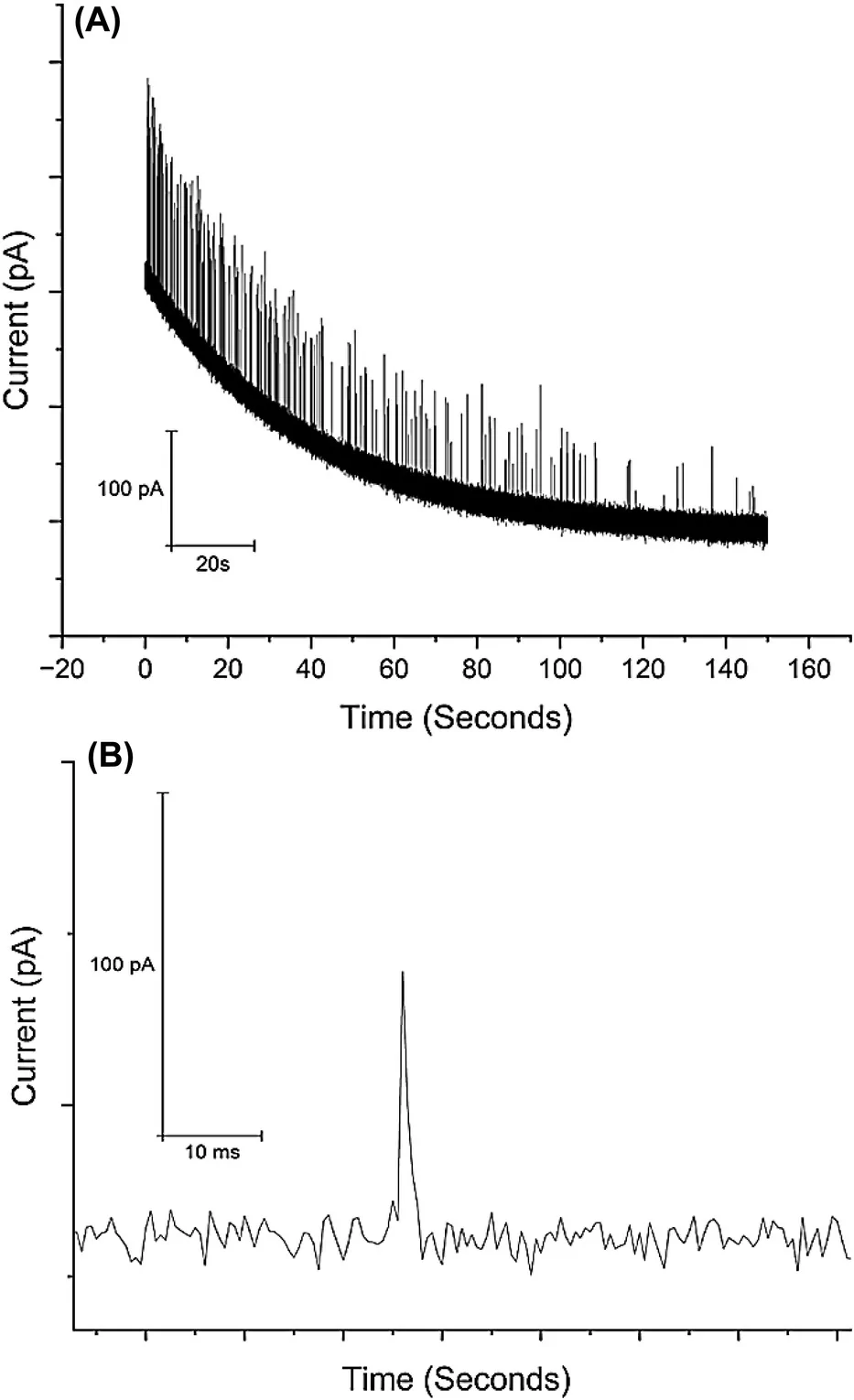
Single entity electrochemical measurement of silver nanoparticles at ~ 80 ± 15 pA, A; 150 s measurement, B; isolated representation of single silver nanoparticle.

On the other hand, modified AgNPs‐MPBA conjugates were also measured in the same way (Fig. 4). In the measurement of silver nanoparticles modified with mercaptophenyl boronic acid with single entity electrochemistry at a height of ~125 ± 15 pA, an increase in peak signals is observed in relatively larger particles, while the increase in the collision frequency (Hz) is small and a relatively 2 ms peak amplitude increase was observed (Fig. 4). To avoid ambiguity, collision frequency is defined here as the number of resolved, independent collision events (peaks separated by the minimum inter‐peak distance) per 150 s trace. MPBA functionalisation does not increase the number of independent collisions; instead, it typically yields higher‐amplitude and broader transients and can lead to a lower resolved collision frequency because particles exhibit longer stays on electrode, which reduces the rate of distinct, separable collision events. Accordingly, GA binding is reflected primarily by event morphology rather than by an increased event rate. This shows that the particle grows and stays on the surface for a longer time. Here, the number of frequencies was determined in the same repeated (n = 10) measurements. Increase in peak heights and reducing the collision frequency were observed.

**Fig. 4:**
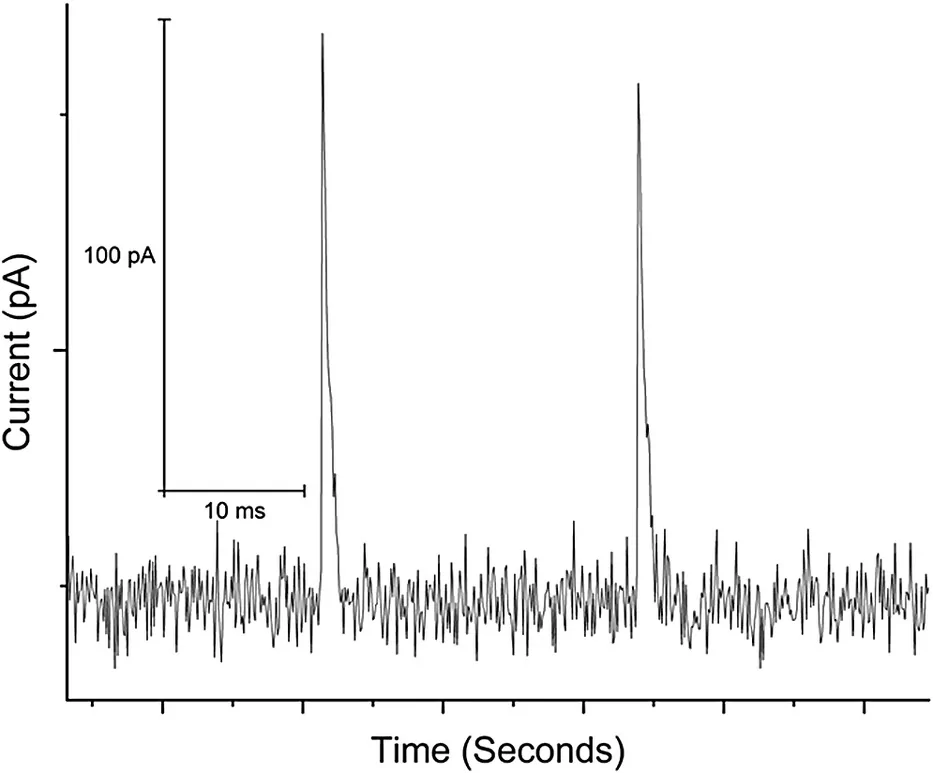
Single entity electrochemistry measurement of mercaptophenyl boronic acid modified silver nanoparticles at ~ 125 ± 15 pA, an isolated demonstration of the sequential collision of single AgNPs‐MPBA.

Afterwards, serum sample was measured with SEE along with glycated products, separately. Figure S3 shows the collisions of all these biomolecules. Such as  phosphate buffer, glucose molecule, glycated albumin, glycated haemoglobin and serum sample. The overlayed data were considered as non‐collision occurrence by comparing Fig. 3. When the height of these possible but not considered as peaks was observed as noise, and an interference effect of 5 pA was also observed. After this stage, the sampling time was increased to 10 ms to reduce this interference effect. Thus, the noise was reduced in smooth and baseline‐corrected data [22]. These incubations were also measured in 3 mL of electrochemical cell. The SEE measurements (*n* = 5) seen in Fig. 3 and the characteristic peaks next to them were defined. Accordingly, when the measurements obtained in order are examined, the AgNPs‐MPBA peak obtained in Fig. 5 (Fig. 5) with glycated‐lysine binding was observed to be ~ 6 ms longer but higher, and again had single peak characteristics with lower collision frequency than AgNPs. Accordingly, an increase in the number of collisions was observed. A relative increase in the peak frequency (number of collisions) was then observed compared to the AgNPs‐MPBA particles incubated with glycated albumin, along with a peak amplitude of ~ 32 ms and a peak height of 285 pA. However, with the increase in particle size and the extension of the residence time on the electrode surface, a secondary, relatively lower signal peak‐tailing was observed after the main peak. At this point, due to the glycated albumin remaining on the surface more than normal, the collision, i.e., the number of electrons given by the nanoparticle as it remains on the surface, also decreases, in other words, a secondary close collision (tailing) was observed with the increase in peak height. The individual peak collision shows the small glycated molecules other than the glycated albumin conjugated with the silver nanoparticle. Here, the GA (Glc‐Alb) characteristic, especially the long peak time and tailing, was a distinguishing feature for measurement.

**Fig. 5:**
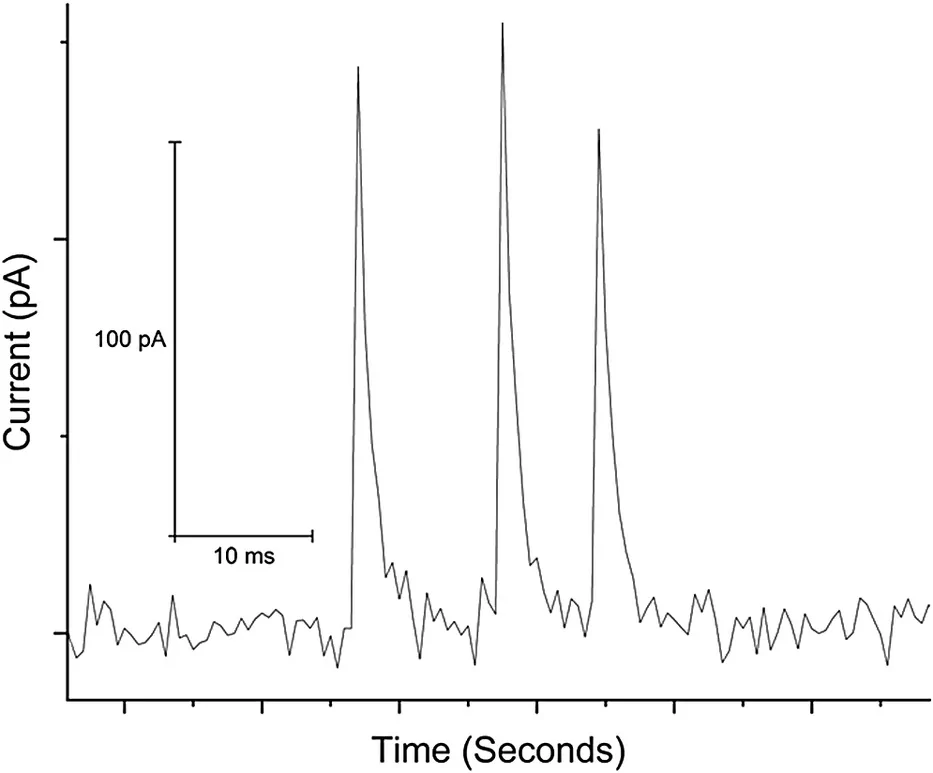
Demonstration of the interaction of glycated‐lysine with its conjugate by single entity electrochemistry, peak current 160 ± 35 pA.

After determining the glycated albumin peak characteristics, optimisation processes were started. The point to be noted here is that instead of comparing the measurements with the data obtained in serum, which is accepted as the baseline, a comparison of AgNPs‐MPBA and AgNPs‐MPBA‐Glc‐Alb, in which the peak characteristics change with each modification and binding, was made. In the AgNPs‐MPBA characteristic peaks obtained (Fig. 4), an increase in peak current, tailing and an increase in the number of collisions with a secondary peak are observed with GA binding (Fig. 6). However, this situation is especially seen when the GA peak characteristics are isolated and examined, and a decrease in the number of collisions is observed (Fig. 7A). Here, the tail character was evaluated as a signal by selecting the peaks with higher peak currents of target collision events using a Python‐integrated rule‐based thresholding within Python workflow [23]. First, the calibration curve was obtained. Accordingly, standard solutions were prepared for GA concentration at the targeted % concentrations in the literature (0.1–50%) and incubated with AgNPs‐MPBA. The SEE data obtained with the standards prepared at the targeted percentage concentrations and the baseline correction of this data are given in Fig. 7. According to the Python code system, only the peaks with tails and at least 32 ms collisions were isolated and used against concentration (Fig. 7B). New formation of the characteristic peak number and frequency increased. In this way, a decrease in standard deviation was targeted along with selectivity (*n* = 5). The calibration graph obtained with the concentration (%) against the collision numbers was given. The sensor system with the conjugate obtained in this way provides a linear measurement in the target range. The collision numbers calculated in Fig. 7C were calculated as 150 s as a frequency. *R* ^2^ was found as 0.9972 ± 0.0025 (*n* = 5). Calibration‐based LOD/LOQ definition using blank standard deviation and calibration slope (LOD = 3.3·σ_blank (frequency of collision)/m; LOQ = 10·σ_blank (frequency of collision)/m), with confirmatory criteria using blank +3σ and blank +10σ and a precision requirement at LOQ. LOD and LOQ were calculated as 0.02% and 0.08%, respectively. Innovative data‐processing steps applied in the analysis of measurement signals (selecting only ‘tailed’ peaks over a certain period) also increased reliability by reducing the standard deviation of the measurements within themselves. The Python‐based analysis developed for this purpose was successful in eliminating noise in the raw data and isolating target signals, thus allowing for clearer measurement of individual collision peaks. This approach stands out as a unique method contributing to the evaluation of single‐entity electrochemistry data in the literature.

**Fig. 6:**
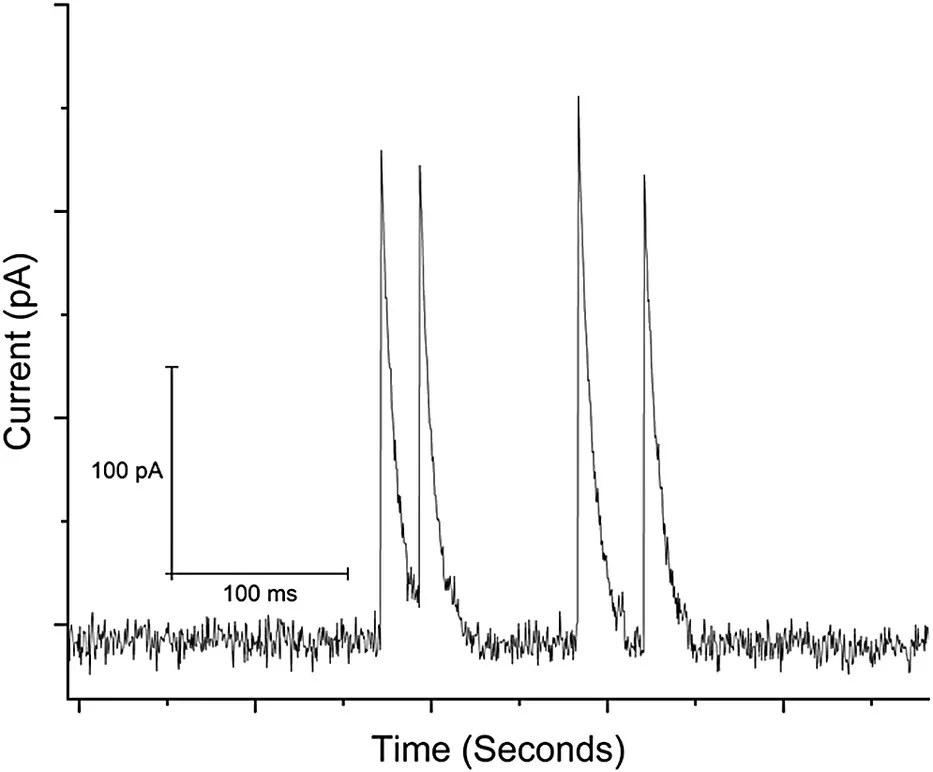
Demonstration of interaction with glycated albumin conjugate by single entity electrochemistry, peak current 285 ± 35 pA.

**Fig. 7:**
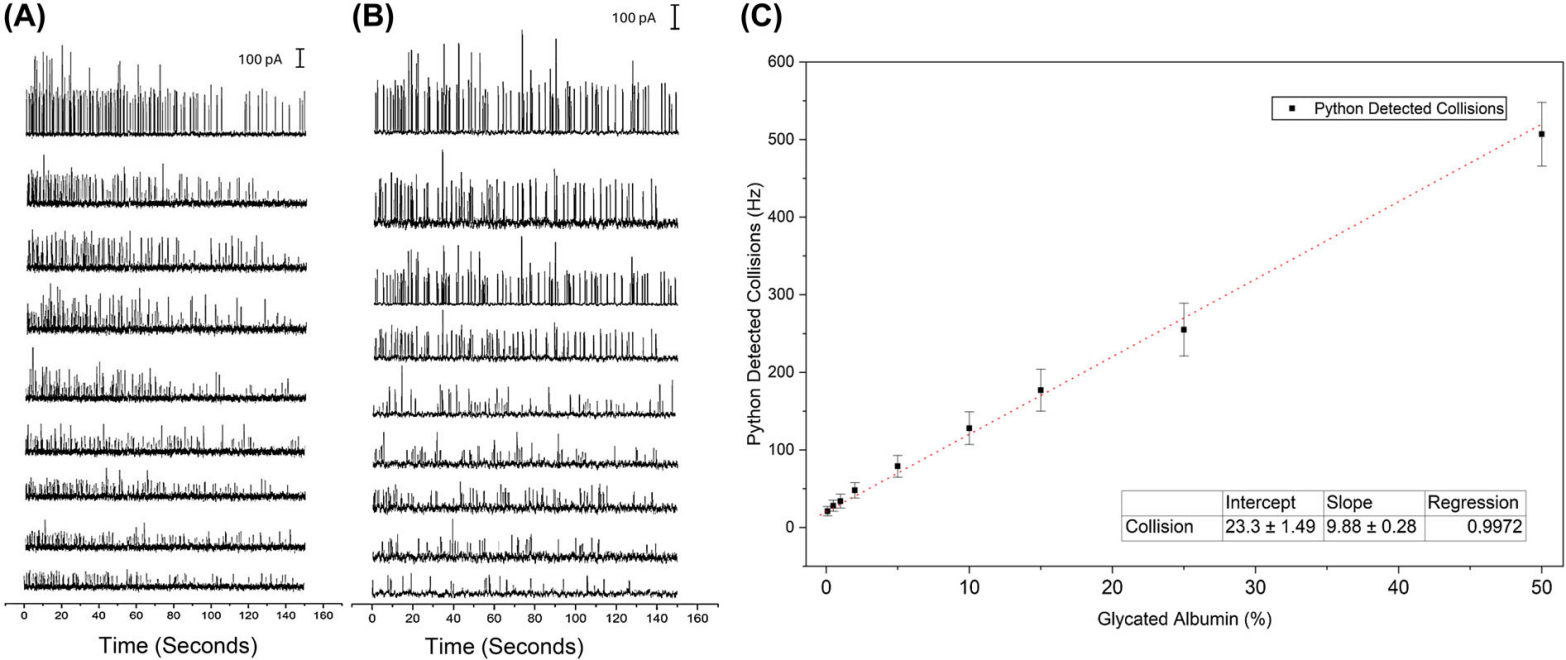
A single‐entity electrochemical analysis of glycated albumin standards from 0.1% to 50%. (A) Baseline‐corrected current‐time responses obtained after incubation of AgNPs‐MPBA with glycated albumin standards. (B) Python‐processed single‐entity responses after baseline correction, smoothing, peak detection, and rule‐based classification. (C) Calibration curve constructed from the number of GA‐classified collision events recorded within a 150 s acquisition window as a function of glycated albumin percentage (error bars represent the standard deviation of five independent measurements).

The scatter shows clear, class‐dependent separation by peak width (*x*) and peak current points (*y*) (Fig. 8). Bare AgNP events cluster at low widths (≈ 3–9 ms) and modest currents (≈ 50–100 pA). AgNP‐MPBA shifts upward and slightly right (≈ 100–160 pA; ≈ 6–12 ms), consistent with surface chemistry, increasing effective size/charge and residence time. Glycated lysine occupies an intermediate band (≈ 140–230 pA; ≈ 12–28 ms). GA (glycated albumin) forms a well‐separated cloud at high current and width (≈ 280–330 pA; ≈ 30–40+ ms), reflecting longer surface residence and the characteristic ‘tailed’ collisions. A simple rule such as width > 30 ms and current > 280 pA would identify GA with minimal overlap, while AgNP vs AgNP‐MPBA are distinguished mainly by current with a narrow width window.

**Fig. 8:**
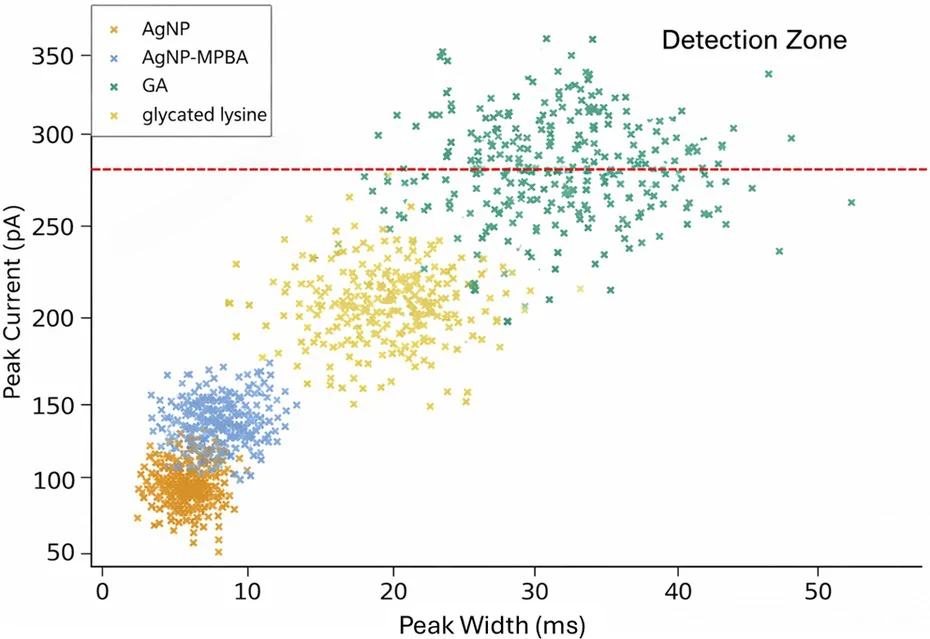
Peak‐shape discrimination against single‐entity collisions in serum and detection zone.

Tailing increases (Fig. S4) monotonically with surface chemistry and GA binding. Bare AgNP events show almost no tailing (~ 0.05), AgNP‐MPBA is still low (~ 0.10–0.12), glycated lysine is intermediate (~ 0.35–0.40), and GA is distinctly high (~ 0.68–0.70). The small error bars (binomial SE) indicate stable estimates across events/replicates. Practically, a simple rule—tailing fraction > 0.5—cleanly flags GA while excluding AgNP and AgNP‐MPBA. Fig. S5 shows the real sample applications with spiked glycated lysine and glycated albumin, Fig. S5A, AgNPs sparse, short events with low currents (~ 50–100 pA) and almost no tailing. B, AgNPs‐MPBA currents shift higher (~ 100–160 pA) with occasional tails—consistent with functionalisation increasing residence time. C, AgNPs‐MPBA + glycated lysine: intermediate regime (~ 150–230 pA), noticeably more tails. D, AgNPs‐MPBA + GA: highest currents (~ 230–350 pA) and frequent long tails, the GA ‘signature’ reported in our method (longer post‐peak decay/width). These qualitative trends align with our SEE settings and classification rules described in the manuscript. Therefore, in the real samples, higher than 230 pA with tail is the indicator of the glycated albumin. Interference testing was performed under high interferent loads using matched serum conditions. The method showed limited susceptibility to common interferents, with bias remaining within approximately ±5% across the panel. Specifically, glucose produced a +1.4% bias, ascorbate −1.0%, urate +2.6%, bilirubin +2.1%, haemolysis −3.2%, and lipaemia +4.5%, indicating that lipaemia was the most influential interferent while remaining within the predefined acceptance range. Overall, these results support that interference is limited for the tested conditions, with severe lipaemia yielding the largest positive bias (≈ +4.5%). In the study, the non‐immobilised, mix‐and‐measure format is a major advantage. Recognition occurs in solution via freely diffusing MPBA‐AgNP conjugates, eliminating surface‐coupling steps that often compromise activity and slow mass transfer. Combined with a < 2 min time‐to‐result and negligible carryover (0.24%), this simplifies workflows and facilitates rapid serial measurements; sensor ‘renewal’ is as simple as introducing a fresh nanoparticle suspension. Interference testing revealed biases within ~ ± 5% under strenuous conditions, with lipaemia producing the largest effect (+4.5%); this can be mitigated by simple clarifications or by flagging highly lipaemic samples for repeat. Remaining limitations include potential batch‐to‐batch variability in nanoparticle properties and electrode surfaces, matrix extremes (severe lipaemia), and the need for larger, multi‐site datasets to further tighten method‐comparison intervals.

## Materials

In this study, pooled human serum samples used as the sample matrix were collected with approval from the Kafkas University Faculty of Medicine Ethics Committee (session dated 29.05.2024; approval no. 437). Serum samples were obtained from volunteers admitted to Kafkas University Research and Education Hospital for routine medical examination. All experiments involving human samples were undertaken with the written and informed consent of each subject. The study methodologies conformed to the standards set by the Declaration of Helsinki. Electrochemical measurements were performed in a three‐electrode configuration using a 25 μm Pt working electrode (MF‐20151), a Pt counter electrode (MW‐1032), and an Ag/AgCl (3.0 M KCl) reference electrode (MF‐2052) (BASi, West Lafayette, IN, USA). All chemical materials were obtained from Sigma Aldrich (Merck KGaA, Darmstadt, Germany). Ivium CompactStat2.h10800 with internal booster was used as the electrochemical analyser (Ivium, Eindhoven, The Netherlands). Iviumsoft 4.1182 software was used as an electrochemical analysis programme. Single entity electrochemistry analysis was performed using coding using OriginPro version 2025 (OriginLab Corporation, Northampton, MA, USA), which can be integrated with Python 3.13 (Python Software Foundation, Wilmington, DE, USA).

## Methods

### Nanoparticle conjugation and characterisation

Prior to performing electrochemical techniques, nanoconjugates were synthesised and characterised. First, AgNPs were produced by chemical reduction procedure using silver nitrate as silver ion source and sodium citrate was used as reducing agent. Specifically, 90 mg of silver nitrate was dissolved in 450 mL of DI and heated to 100 °C under rapid stirring (500 rpm) with overhead stirrer in a 1 L three‐neck round‐bottomed flask (Chemglass) [24]. The temperature was maintained by reflux condenser. Then, 10 mL of 1% Na Citrate solution was added and the reaction was continued for another 30 min. Afterwards, it was cooled to room temperature and stored in a bottle to determine electrochemical oxidation potential and particle sizes by DLS. Then silver nanoparticles were modified with 4‐mercaptophenylboronic acid (4‐MPBA) [25]. First, 50 μL of 1% Tween 20 was added into 600 μL of AgNPs. Thereupon, 6 μL of 10 μm 4‐MPBA solution and 2 μL of 500 mm pH = 3 citrate buffer was added and shaken for 15 min at 350 rpm. 6 μL of 500 mm pH = 6.5 HEPES buffer was added on this mixture, 2 more μL of 4‐MPBA was added and incubated at 4 °C for 12 h. After incubation, 900 μL of deionised water (DI) was added and centrifuged at 8000 g for 20 min, the upper phase was removed and 900 μL of DI was added and centrifuged twice at 6000 g. This modification was measured by FTIR. Then, glycated albumin, glycated lysine and purchased HbA1c were obtained as simulated. 50 mg/mL stock albumin solution and 30 mg/mL glucose solution were prepared and mixed in 0.1 m phosphate buffer at pH = 7.4. It was then shaken at low speed at 37.5 °C for three weeks. At the end all configurations were confirmed by FTIR again. The Glc‐Albumin complex was then transferred to the dialysis tube and dialysed in at least 1 L phosphate buffer in a cold environment overnight to remove unbound glucose from the medium. All obtained samples were stored in 100 μL aliquoted at −20 degrees until used.

#### Electrochemical measurements

As a triple electrode system, micro platinum electrodes, silver/silver chloride reference electrode and platinum auxiliary electrode were used as mentioned in the materials section. Before the tests of the prepared particles with macroelectrodes, the reduction and oxidation potentials will be determined in buffers prepared at different pHs (pH = 4, 7 and 9). Then, oxidation potential was found between −0.2 and + 0.6 V by cyclic voltammetry. The reason for this process was to determine the potential to be applied for the chronoamperometric method to be applied in single entity electrochemistry. Single particle collision electrochemical (SPCE) measurements of AgNPs distributions (standards) were performed by pipetting 100, 200 or 300 μL of AgNPs stock solution into the electrochemical cell. It was aimed at seeing a current at pA level for the nanoparticles tested in the electrochemical cell containing 5 mL pH = 7.4 phosphate buffer. AgNPs, 4‐MPBA conjugated with AgNPs, and glycated albumin, lysine and HbA1c were added to the medium and the collision numbers to the microelectrode were calculated. The peak characteristics obtained here constitute the main method of measurement. With this calculation, the collision numbers were calculated as the free silver nanoparticles conjugated over time. By this decrease, the amount of glycated albumin was determined by the decrease in chronoamperometric signals.

### Python workflow

Electrochemical current‐time traces were processed using a compact Python‐based workflow designed for baseline extraction, peak detection, and event classification. Only essential libraries were used in order to maintain a lightweight and reproducible analysis environment. NumPy and pandas were used for numerical operations and data handling, Matplotlib was used only for visualisation, and SciPy was used for exponential baseline fitting and peak detection functions. No deep‐learning or other heavy external libraries were included. Analysis parameters were organised in a dedicated settings data class, in which each parameter was explicitly defined together with its type and default value. This structure also included CSV import settings compatible with European formatting conventions, including semicolon‐separated columns and comma decimal notation. For baseline correction, a reference AgNPs‐MPBA signal could be supplied by the user and used as a common baseline for all glycated albumin‐containing samples. When no reference baseline was provided, the baseline of each file was determined individually from the initial 20 s of the signal. Data files were imported using a CSV‐reading function, and baseline behaviour was modeled using a three‐parameter exponential decay function. Baseline fitting was performed on the initial part of the signal, and the mean deviation (μ) and standard deviation (σ) from the fitted model were calculated. These values were subsequently used to define the threshold for peak detection. To improve signal quality, the traces were smoothed using a second‐order five‐point Savitzky–Golay filter, corresponding to an effective window of approximately 25 ms at a sampling interval of 0.005 s. This filtering step suppressed low‐level noise while preserving transient AgNP collision peaks. Peak detection was performed using the SciPy find_peaks function. The detection threshold was defined relative to the baseline noise level, and the minimum distance between neighbouring peaks was set to approximately 20 ms, converted to the corresponding number of sampling points according to the acquisition interval. For each detected event, the tail length was calculated as the time elapsed from the peak maximum until the signal amplitude decayed to 5% of its peak value. This approach enabled adaptive and peak‐specific characterisation of transient electrochemical events. Classification was then performed on the basis of event width using a rule‐based function in which peaks were assigned to predefined classes corresponding to naked AgNPs, MPBA‐modified AgNPs, glucose‐bound species, glycated lysine‐bound species, and glycated albumin‐bound species. Each processed file yielded a peak table containing file name, peak time, amplitude, tail width, and assigned class label. When visualisation mode was enabled, the signal trace and classified peaks were displayed graphically using class‐specific colour annotations for visual inspection. Batch processing was performed automatically for all CSV files in a selected folder, and the extracted peak data from all files were combined into a single dataset. The command‐line workflow also allowed the user to define the data directory, an optional reference baseline file, and the output file name. The final classified output was written as a CSV file. This workflow converted the physical characteristics of experimental single‐entity electrochemical signals into transparent mathematical classification rules, enabling reproducible and interpretable analysis of large datasets containing hundreds to thousands of collision events.

## Conflicts of interest

The author declares no conflicts of interest.

## Author contributions

ZOU: methodology, writing, experimenting, sample collection, writing of original draft, data acquisition, funding acquisition.

## Supporting information


**Fig. S1.** Illustration of the analysis of produced nanoparticles by DLS and particle size distribution.
**Fig. S2.** High‐magnification SEM (InLens, 2.0 kV; WD 4.8 mm; 75 000×) of citrate‐reduced AgNPs on carbon substrate.
**Fig. S3.** Measurement of glycosylated products, serum and buffer by single‐entity electrochemistry.
**Fig. S4.** Tailing frequency by classification. Fraction of single‐entity collision peaks exhibiting post‐peak tailing for AgNP, AgNP‐MPBA, glycated lysine, and GA (150 s per replicate; points = mean; error bars = binomial SE).
**Fig. S5.** Real‐sample single‐entity collisions in serum: (A) AgNPs in serum, (B) AgNPs‐MPBA, (C) AgNPs‐MPBA + glycated lysine, and (D) AgNPs‐MPBA + GA.
**Code S1.** Python workflow for baseline correction, smoothing, peak detection, tail‐width calculation, and classification of single‐entity electrochemical signals.

## Data Availability

The data that support the findings of this study are available in the article and its Supporting Information. Additional raw electrochemical data and Python analysis outputs are available from the corresponding author upon reasonable request.

## References

[1] Allerston LK & Rees NV (2018) Nanoparticle impacts in innovative electrochemistry. Curr Opin Electrochem 10, 31–36.

[2] Zhang L , Wahab OJ , Jallow AA , O'Dell ZJ , Pungsrisai T , Sridhar S , Vernon KL , Willets KA & Baker LA (2024) Recent developments in single‐entity electrochemistry. Anal Chem 96, 8036–8055. 10.1021/acs.analchem.4c01406 PMC11112546 38727715

[3] Chen CH , Ravenhill ER , Momotenko D , Kim YR , Lai SCS & Unwin PR (2015) Impact of surface chemistry on nanoparticle–electrode interactions in the electrochemical detection of nanoparticle collisions. Langmuir 31, 11932–11942. 10.1021/acs.langmuir.5b03033 26448140

[4] Liu J , Ma C , Shi S , Liu H , Wen W , Zhang X , Wu Z & Wang S (2022) A general controllable release amplification strategy of liposomes for single‐particle collision electrochemical biosensing. Biosens Bioelectron 207, 114182. 10.1016/j.bios.2022.114182 PMC8925861 35305388

[5] Liu M , Zhao X , Liang X & Zhou YG (2024) Homogeneous and label‐free detection and monitoring of protein kinase activity using the impact electrochemistry of Silver nanoparticles. ACS Sens 9, 110–117. 10.1021/acssensors.3c01703 38113272

[6] Castañeda AD , Brenes NJ , Kondajji A & Crooks RM (2017) Detection of microRNA by electrocatalytic amplification: a general approach for single‐particle biosensing. J Am Chem Soc 139, 7657–7664. 10.1021/jacs.7b03648 28537750

[7] Ko E , Tran VK , Geng Y , Kim MK , Jin GH , Son SE , Hur W & Seong GH (2018) Determination of glycated albumin using boronic acid‐derived agarose beads on paper‐based devices. Biomicrofluidics 12, 1–8. 10.1063/1.5021395 PMC5796828 29464009

[8] Thapa M & Heo YS (2025) Label‐free electrochemical biosensor for glycated hemoglobin (HbA1c) and glycated albumin (GA) measurement using 4‐mercapto‐phenyl boronic acid embedded affinity. Biochip J 19, 248–261.

[9] Colberg SR , Sigal RJ , Yardley JE , Riddell MC , Dunstan DW , Dempsey PC , Horton ES , Castorino K & Tate DF (2016) Physical activity/exercise and diabetes: a position statement of the American Diabetes Association. Diabetes Care 39, 2065–2079. 10.2337/dc16-1728 PMC6908414 27926890

[10] Saudek CD , Derr RL & Kalyani RR (2006) Assessing glycemia in diabetes using self‐monitoring blood glucose and hemoglobin A1c. JAMA 295, 1688–1697. 10.1001/jama.295.14.1688 16609091

[11] Freitas PAC , Ehlert LR & Camargo JL (2017) Glycated albumin: a potential biomarker in diabetes. Arch Endocrinol Metab 61, 296–304. 10.1590/2359-3997000000272 PMC10118799 28699985

[12] Ki H , Oh J , Han GR & Kim MG (2020) Glycation ratio determination through simultaneous detection of human serum albumin and glycated albumin on an advanced lateral flow immunoassay sensor. Lab Chip 20, 844–851. 10.1039/c9lc00967a 31984402

[13] Ran G , Chen X & Xia Y (2017) Electrochemical detection of serotonin based on a poly(bromocresol green) film and Fe3O4 nanoparticles in a chitosan matrix. RSC Adv 7, 1847–1851.

[14] Pagana KD , Pagana TJ & Pagana TN (2024) Mosby's Diagnostic and Laboratory Test Reference. Elsevier, St. Louis.

[15] Kohzuma T , Yamamoto T , Uematsu Y , Shihabi ZK & Freedman BI (2011) Basic performance of an enzymatic method for glycated albumin and reference range determination. J Diabetes Sci Technol 5, 1455–1462. 10.1177/193229681100500619 PMC3262714 22226265

[16] Silver AC , Lamb E , Cattell WR & Dawnay ABSJ (1991) Investigation and validation of the affinity chromatography method for measuring glycated albumin in serum and urine. Clin Chim Acta 202, 11–22. 10.1016/0009-8981(91)90251-7 1807865

[17] Panja A , Mishra AK , Dash M , Pandey NK , Singh SK & Kumar B (2021) Silver nanoparticles – a review. Eurasian J Med Oncol 5, 95–102.

[18] Onur Uygun Z & Ertuğrul Uygun HD (2020) A novel Chronoimpedimetric glucose sensor in real blood samples modified by glucose‐imprinted pyrrole‐Aminophenylboronic acid modified screen printed electrode. Electroanalysis 32, 226–229.

[19] Uygun ZO & Tasoglu S (2024) Impedimetric antimicrobial peptide biosensor for the detection of human immunodeficiency virus envelope protein gp120. iScience 27, 109190. 10.1016/j.isci.2024.109190 PMC10897921 38414859

[20] Ezer M & Uygun ZO (2025) Python‐driven impedance profiling on peptide‐functionalized biosensor for detection of HIV gp41 envelope protein. 3 Biotech 15, 229. 10.1007/s13205-025-04400-8 PMC12209138 40599495

[21] Navolotskaya DV , Toh HS , Batchelor‐McAuley C & Compton RG (2015) Voltammetric study of the influence of various phosphate anions on Silver nanoparticle oxidation. ChemistryOpen 4, 595. 10.1002/open.201500100 PMC4608526 26491638

[22] Savitzky A & Golay MJE (2002) Smoothing and differentiation of data by simplified least squares procedures. Anal Chem 36, 1627–1639. 10.1021/ac60319a045 22324618

[23] Zhao Z , Naha A , Ganguli S & Sekretareva A (2023) Automated Analysis of Nano‐Impact Single‐Entity Electrochemistry Signals using Unsupervised Machine Learning and Template Matching. Adv Intell Syst 6, 2300424.

[24] Mikac L , Ivanda M , Gotić M , Mihelj T & Horvat L (2014) Synthesis and characterization of silver colloidal nanoparticles with different coatings for SERS application. J Nanopart Res 16, 1–13.

[25] Weiß LJK , Nikić M , Simmel FC & Wolfrum B (2025) Stochastic impact electrochemistry of Alkanethiolate‐functionalized Silver nanoparticles. Small 21, 2410306. 10.1002/smll.202410306 PMC12019921 40079073

