# Supplementary material for: Ultrafast python‐integrated single‐entity electrochemical sensor for detecting glycated albumin
Source: FEBS J. 2026 Apr 18;293(19):5898–907. doi: 10.1111/febs.70547 (PMC13628537; doi:10.1111/febs.70547)
Supplement: Supplementary file 1 [file FEBS-293-5898-s001.pdf]

SUPPLEMENTARY MATERIAL

Ultrafast Python-integrated sensor based on nanocollision for detection of glycated albumin in real samples

Zihni Onur Uygun*

Kafkas University, Faculty of Medicine, Department of Medical Biochemistry, Kars, Türkiye

*Corresponding author: onur.uygun@kafkas.edu.tr

Size d.nm	Mean Intensity Percent	Std Dev Intensity Percent
0,4000	0,0	
0,4632	0,0	
0,5365	0,0	
0,6213	0,0	
0,7195	0,0	
0,8332	0,0	
0,9649	0,0	
1,117	0,0	
1,294	0,0	
1,499	0,0	
1,736	0,0	
2,010	0,0	
2,328	0,0	
2,696	0,0	
3,122	0,0	
3,615	0,0	
4,187	0,0	
4,849	0,0	
5,615	0,0	
6,503	0,0	
7,531	0,1	
8,721	0,3	
10,10	0,5	
11,70	0,6	

Size d.nm	Mean Intensity Percent	Std Dev Intensity Percent
13,54	0,6	
15,69	0,5	
18,17	0,4	
21,04	0,4	
24,36	0,6	
28,21	1,1	
32,67	1,9	
37,84	3,0	
43,82	4,4	
50,75	5,9	
58,77	7,3	
68,06	8,4	
78,82	9,3	
91,28	9,6	
105,7	9,5	
122,4	8,9	
141,8	7,8	
164,2	6,5	
190,1	5,0	
220,2	3,5	
255,0	2,1	
295,3	1,1	
342,0	0,4	
396,1	0,1	

Size d.nm	Mean Intensity Percent	Std Dev Intensity Perce
458,7	0,0	
531,2	0,0	
615,1	0,0	
712,4	0,0	
825,0	0,0	
955,4	0,0	
1106	0,0	
1281	0,0	
1484	0,0	
1718	0,0	
1990	0,0	
2305	0,0	
2669	0,0	
3091	0,0	
3580	0,0	
4145	0,0	
4801	0,0	
5560	0,0	
6439	0,0	
7456	0,0	
8635	0,0	
1,000e4	0,0	

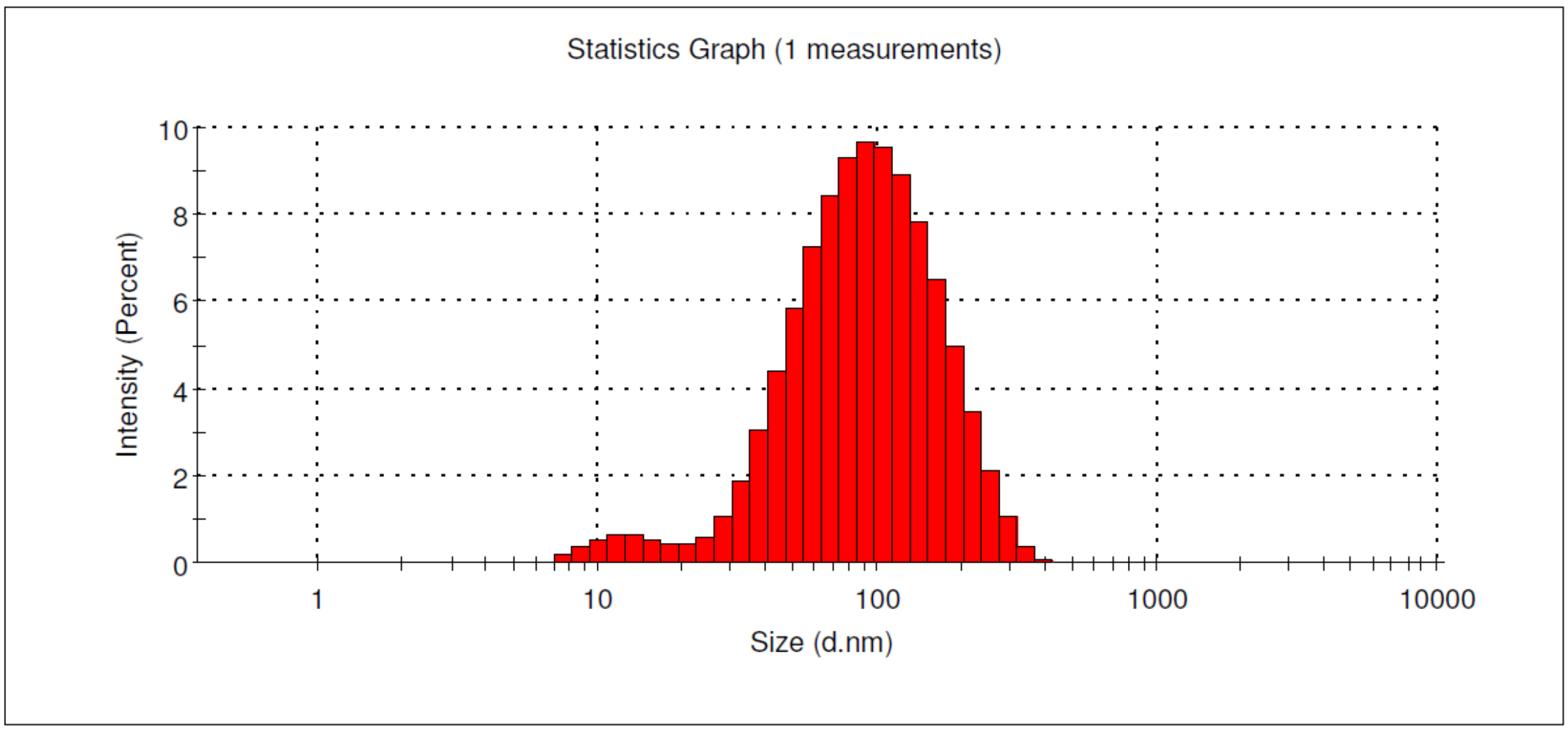

Supplementary figure 1. Illustration of the analysis of produced nanoparticles by DLS and particle size distribution.

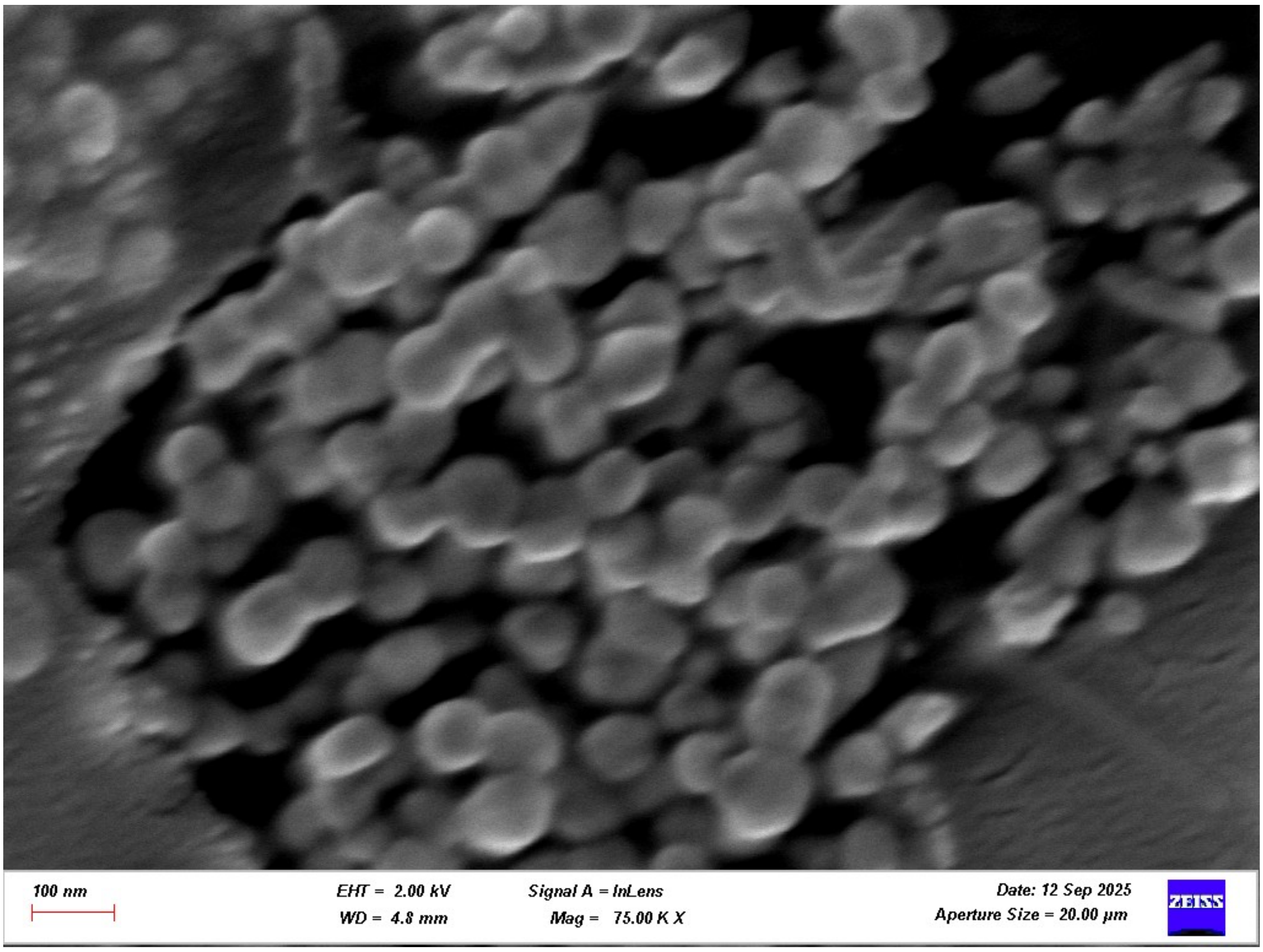

Supplementary figure 2. High-magnification SEM (InLens, 2.0 kV; WD 4.8 mm; 75,000×) of citrate-reduced AgNPs on carbon substrate.

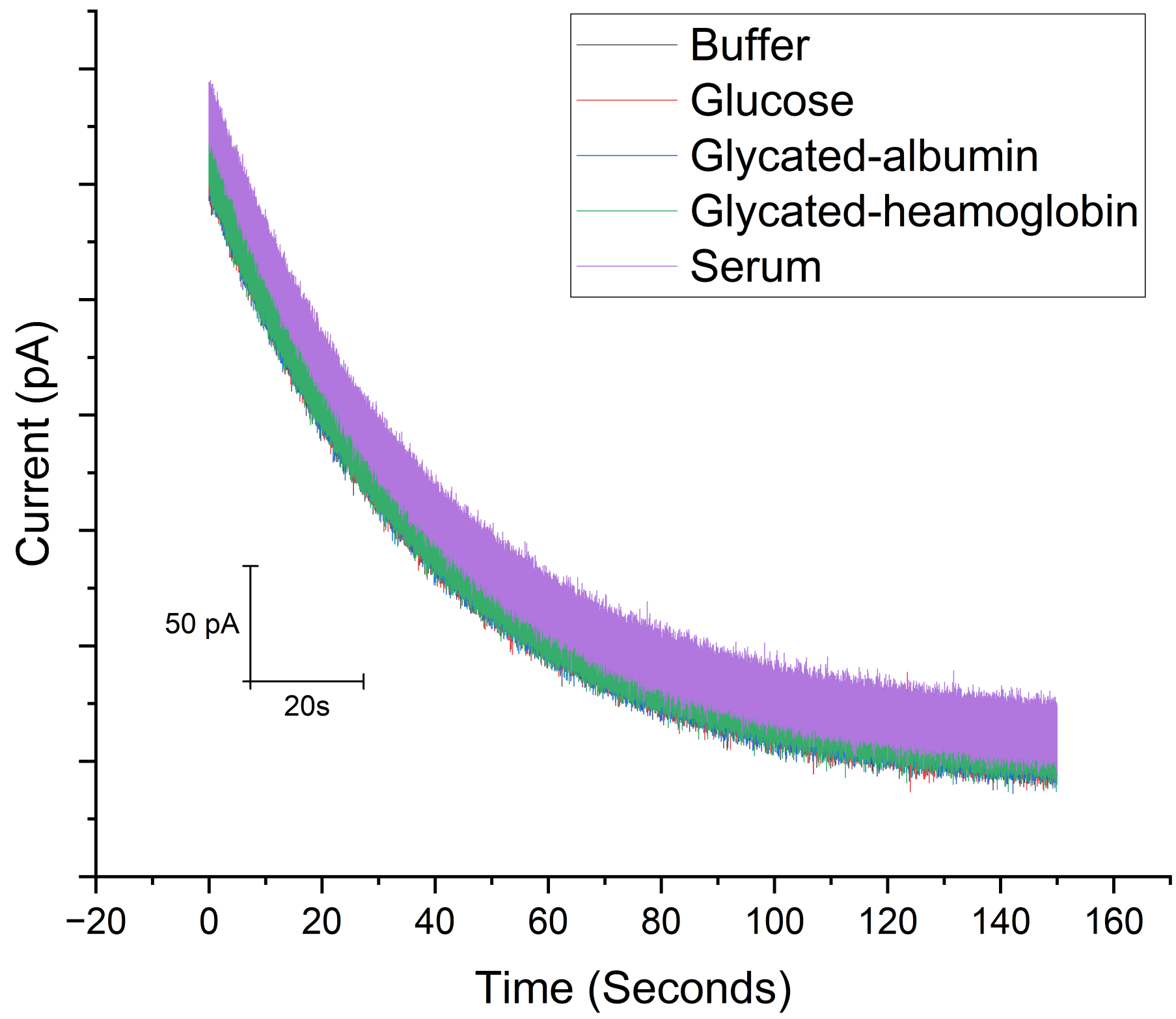

Supplementary figure 3. Measurement of glycosylated products, serum and buffer by single entity electrochemistry

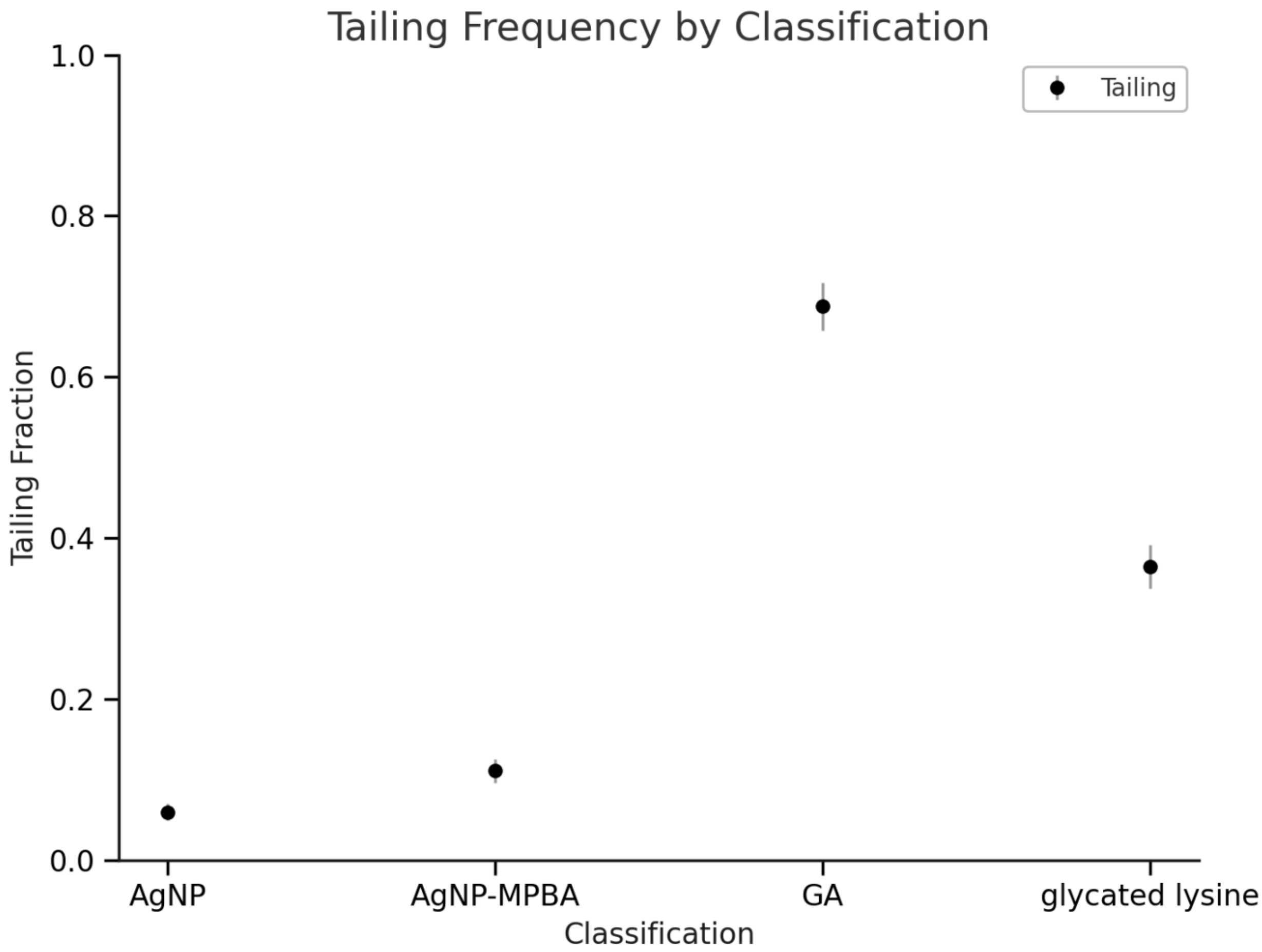

Supplementary figure 4. Tailing frequency by classification. Fraction of single-entity collision peaks exhibiting post-peak tailing for AgNP, AgNP-MPBA, glycated lysine, and GA (150 s per replicate; points = mean; error bars = binomial SE).

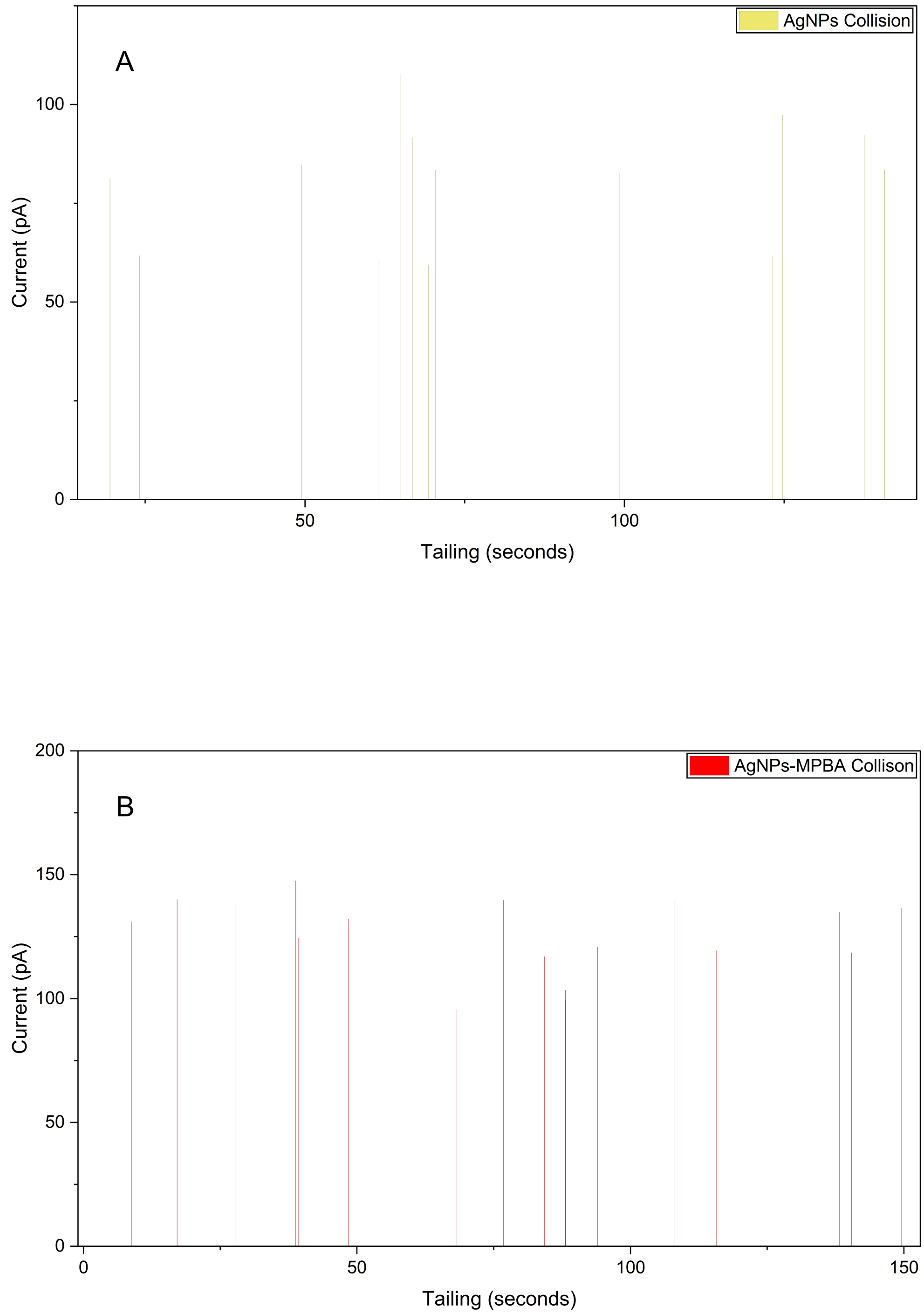
AgNPs Collision
A
Current (pA)
Tailing (seconds)
0
50
100
AgNPs-MPBA Collison
B
150
200

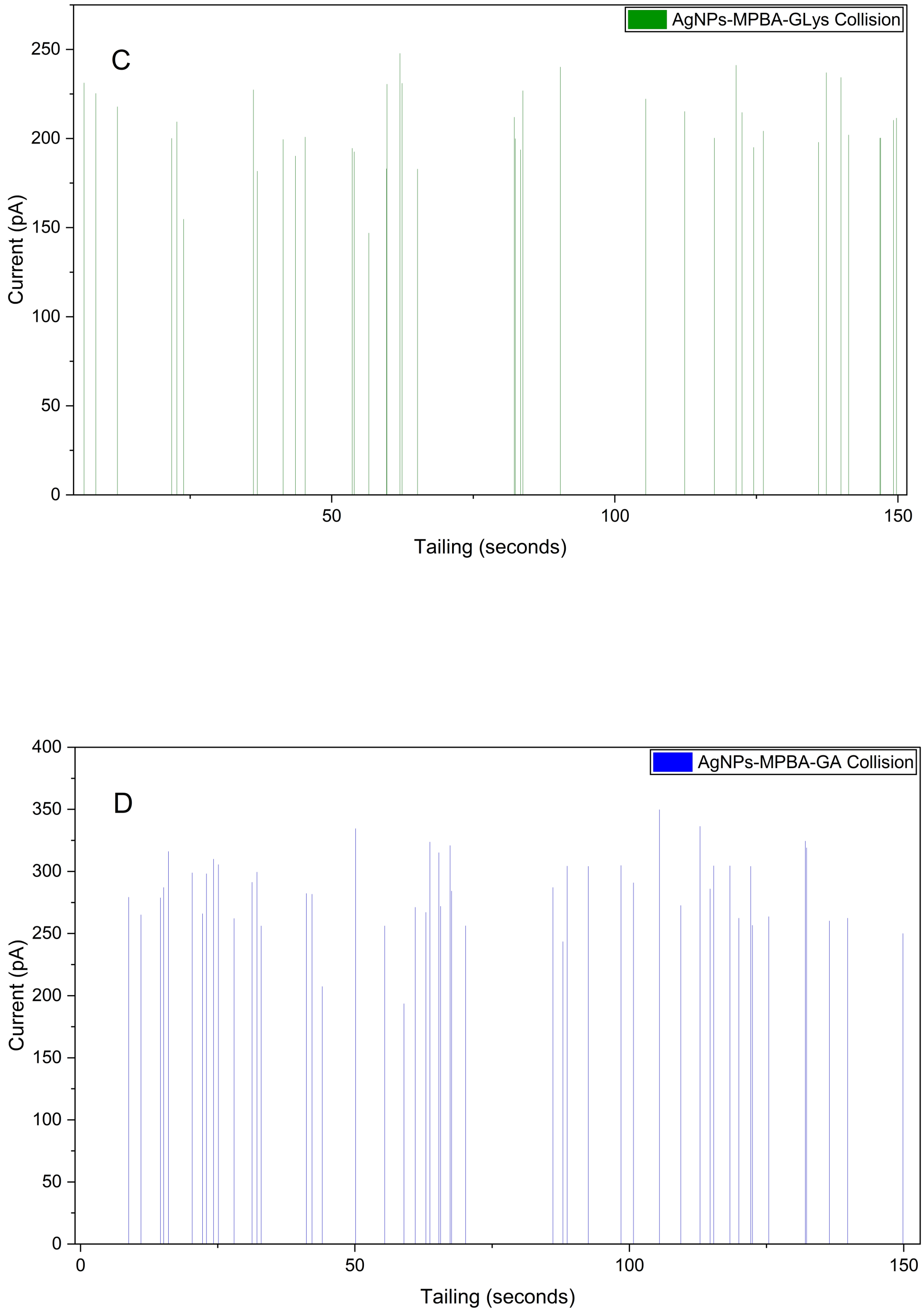

Supplementary figure 5. Real-sample single-entity collisions in serum, (A) AgNPs in serum, (B) AgNPs-MPBA, (C) AgNPs-MPBA + glycated lysine, (D) AgNPs-MPBA + GA.

Python Codes

```
# see_originpro_classifier.
import os
import numpy as np
import pandas as pd
import matplotlib.pyplot as plt
from scipy.signal import find_peaks, savgol_filter
from scipy.optimize import curve_fit

# === USER CONFIGURATION ===
INPUT_FOLDER = "C:/SEE_data"
OUTPUT_FILE = "C:/SEE_data/classified_peaks.csv"
BASELINE_FILE = None  # example: "C:/SEE_data/baseline.csv"
DRAW_PLOTS = True
SAMPLING_INTERVAL = 0.005  # s

# === PEAK DETECTION SETTINGS ===
TAIL_THRESHOLD = 0.005
NOISE_SIGMA_MULTIPLE = 4
MIN_PEAK_DISTANCE_S = 0.040
SG_WINDOW = 5
SG_POLY = 2
BASELINE_WINDOW_S = 20.0

# === CLASSIFICATION THRESHOLDS (by peak width in seconds) ===
# NOTE: Updated label "Glc" -> "Gly-Lys" (glycated lysine)
WIDTH_THRESHOLDS = {
    "AgNP": 0.004,
    "MPBA": 0.007,
    "Gly-Lys": 0.025,
    "Gly-Alb": 0.032  # ≥ this value
}

def read_csv_origin(path):
    # European format: semicolon separator, comma decimal point
    return pd.read_csv(path, sep=";", decimal=",")
```

```
def exp_decay(t, i_inf, i0, k):
    return i_inf + (i0 - i_inf) * np.exp(-k * t)

def fit_baseline(t, i):
    mask = t <= BASELINE_WINDOW_S
    p0 = (i[mask].min(), i[mask].max(), 0.02)
    popt, _ = curve_fit(exp_decay, t[mask], i[mask], p0=p0, maxfev=10000)
    baseline = exp_decay(t, *popt)
    residual = i[mask] - exp_decay(t[mask], *popt)
    return baseline, residual.mean(), residual.std(ddof=1)

def baseline_subtract(t, i, baseline_data=None):
    if baseline_data is not None:
        t_base, i_base = baseline_data
        baseline = np.interp(t, t_base, i_base)
        mu = 0.0
        sigma = np.std(i_base[:int(BASELINE_WINDOW_S / SAMPLING_INTERVAL)])
    else:
        baseline, mu, sigma = fit_baseline(t, i)
    return i - baseline - mu, sigma

def classify_width(w):
    # class based on ascending width thresholds
    for cls, w_max in WIDTH_THRESHOLDS.items():
        if w < w_max:
            return cls
    return "Unknown"

def tail_end(y, start_idx):
    thr = y[start_idx] * TAIL_THRESHOLD
    k = start_idx
    while k < len(y) and y[k] > thr:
        k += 1
    return k
```

```
def process_file(filepath, baseline_ref=None):
    df = read_csv_origin(filepath)
    t = df.iloc[:, 0].astype(float).to_numpy()
    i = df.iloc[:, 1].astype(float).to_numpy()

    i_corr, sigma = baseline_subtract(t, i, baseline_ref)
    i_filt = savgol_filter(i_corr, SG_WINDOW, SG_POLY)

    peaks, _ = find_peaks(
        i_filt,
        height=NOISE_SIGMA_MULTIPLE * sigma,
        distance=int(MIN_PEAK_DISTANCE_S / SAMPLING_INTERVAL)
    )

    records = []
    for p in peaks:
        end = tail_end(i_filt, p)
        width = (end - p) * SAMPLING_INTERVAL
        records.append({
            "file": os.path.basename(filepath),
            "apex_time_s": float(t[p]),
            "amp_A": float(i_filt[p]),
            "width_s": float(width),
            "class": classify_width(width)
        })

    # Optional plot
    if DRAW_PLOTS:
        plt.figure(figsize=(10, 3))
        plt.plot(t, i_filt, lw=0.4, label="filtered")

        # Updated: "Gly-Lys" instead of "Glc"
        for cls, color in [("AgNP", "black"),
                           ("MPBA", "blue"),
                           ("Gly-Lys", "green"),
                           ("Gly-Alb", "red")]:
```

```
            m = [r for r in records if r["class"] == cls]
            if m:
                times = [r["apex_time_s"] for r in m]
                amps = [r["amp_A"] for r in m]
                plt.scatter(times, amps, s=10, label=cls, c=color)

        plt.title(os.path.basename(filepath))
        plt.xlabel("Time (s)")
        plt.ylabel("Current (A)")
        plt.legend()
        plt.tight_layout()
        plt.show()

    return records

def load_baseline():
    if BASELINE_FILE is None:
        return None
    df = read_csv_origin(BASELINE_FILE)
    t = df.iloc[:, 0].astype(float).to_numpy()
    i = df.iloc[:, 1].astype(float).to_numpy()
    return (t, i)

# === MAIN EXECUTION ===
all_records = []
baseline_data = load_baseline()

for fname in sorted(os.listdir(INPUT_FOLDER)):
    if fname.lower().endswith(".csv"):
        path = os.path.join(INPUT_FOLDER, fname)
        recs = process_file(path, baseline_data)
        all_records.extend(recs)

# Export results
df_out = pd.DataFrame(all_records)
df_out.to_csv(OUTPUT_FILE, index=False, sep=",", decimal=".", encoding="utf-8-sig")
```

```
print(f"{len(df_out)} peaks classified. Saved to: {OUTPUT_FILE}")
```
